# Effects of Boiling Processing on Texture of Scallop Adductor Muscle and Its Mechanism

**DOI:** 10.3390/foods11131947

**Published:** 2022-06-30

**Authors:** Zi-Xuan Wu, Ying-Chen Fan, Chao Guo, Yu-Xin Liu, De-Yang Li, Peng-Fei Jiang, Lei Qin, Yan-Hong Bai, Da-Yong Zhou

**Affiliations:** 1 National Engineering Research Center of Seafood, Dalian 116034, China; zixuanwu_0609@163.com (Z.-X.W.); fanyingchen723@163.com (Y.-C.F.); gc823705522@163.com (C.G.); forever--xin@126.com (Y.-X.L.); dpuldy@163.com (D.-Y.L.); 13840940070@163.com (P.-F.J.); qinlei@dlpu.edu.cn (L.Q.); 2 School of Food Science and Technology, Dalian Polytechnic University, Dalian 116034, China; 3 College of Food and Bioengineering, Zhengzhou University of Light Industry, Zhengzhou 450002, China; baiyanhong212@163.com

**Keywords:** scallop, boiling, texture, protein denaturation, protein degradation, muscle fiber, myofibril

## Abstract

The objective of this study was to reveal the effects of boiling processing on the texture of scallop adductor muscle (SAM) and its mechanism. Compared to the fresh sample, all the texture indicators, including the hardness, chewiness, springiness, resilience, cohesiveness, and shear force of 30-s- and 3-min-boiled SAMs increased time-dependently (*p* < 0.05). As the boiling time increased further to 15 min, the shear force and cohesiveness still increased significantly (*p* < 0.05), and the resilience and hardness were maintained (*p* > 0.05), but the springiness and chewiness decreased significantly (*p* < 0.05). The overall increase in the texture indicators of the boiled SAMs was due to the boiling-induced protein denaturation, aggregation, and increased hydrophobicity, resulting in the longitudinal contraction and lateral expansion of myofibrils, the longitudinal contraction and lateral cross-linked aggregation of muscle fibers, and the loss of free water. However, the decreasing springiness and chewiness of the 15-min-boiled SAMs was due to the significant degradation of proteins (especially collagen), resulting in the destruction of the connective tissue between the muscle fiber clusters. Both from a subjective sensory point of view and from the objective point of view of protein denaturation and degradation, 3-min-boiled SAMs are recommended. The quality improvement of thermally processed products by controlled, moderate cooking is of practical value from the perspective of food consumption.

## 1. Introduction

Scallop is a kind of bivalve with important commercial value, and its global output was more than 2.8 million tons in 2020 [1]. As a popular edible marine mollusk, scallop consumption is on the rise, owing to its distinctive flavor and delicious taste, as well as its health-beneficial substances, such as valuable peptides, n-3 polyunsaturated fatty acids, vitamins, and other bioactive compounds [2]. In addition to direct fresh consumption, scallop can also be processed into dried products, ready-to-eat products, frozen products, canned products, and so on, resulting in increasing market demand.

Fresh seafood is prone to corruption due to endogenous proteases and environmental microbes. Thus, it is difficult to transport or store for long period. A wide range of thermal processing methods (such as boiling, steaming, baking, roasting, grilling, broiling, braising, frying/deep frying, etc.) have been employed to ensure the safety and extend the shelf life of seafood products [3]. Of these which is a necessary step in industrial processing of dried, semi-cooked and ready-to-eat seafood products, is the most commonly used. The selection of optimal boiling processing conditions for aquatic muscle foods is important for their sensory qualities, especially their texture. Generally, moderately boiled aquatic muscle food has the high-quality texture characteristics of moderate tenderness and good elasticity. Insufficient boiling usually impoverishes the texture of aquatic muscle food. For example, in marine gastropods, the disruption of the collagen conformation at the initial stage of boiling results in significant shrinkage and toughening [4,5]. However, excessive boiling results in texture deterioration (the product becomes soft due to a loss of elasticity and tensile force) for aquatic muscle food, which is known for its sensitivity to high thermal load [3]. Currently, the precise judgment basis for suitable heating conditions is largely unknown. According to a previous study [6], suitable heating conditions for scallop adductor muscles (SAMs) as a kind of muscle food can be developed by monitoring the denaturation of protein. Differential scanning calorimetry (DSC) enables quick, convenient, and precise measurements on the denaturation of muscle protein directly in situ through its thermal behavior [6,7]. This apparatus can be used to determine whether aquatic muscle food is cooked completely.

The effects of thermal processing on the texture properties of scallops have also been investigated. Abe and Miyashita [8] found that the hardness of scallop (*Patinopecten yessoensis*) adductor muscle increased during steaming at increasing temperatures of 98, 150, and 200 °C, respectively, which may be due to increasing water loss, as well as the observed muscular fiber bunching. Dong et al. [9] found that the shear force and hardness of scallop (*Patinopecten yessoensis*) adductor muscle increased first and then decreased during thermal processing at 55 °C for 32 h, and they speculated that these changes in texture were related to the low-temperature, long-term inducement of ROS production, protein oxidation, and degradation of structural proteins. However, the systematic mechanism behind the effects of boiling on the texture properties of scallops is still unknown.

Among the several edible species of scallops, *Chlamys farreri* is one of the most important species found along the coasts of China, Japan, and North Korea [10]. Since there are few reports focused on the texture of *Chlamys farreri* during heat treatment, we chose it as the research subject. The purpose of this study was to monitor protein denaturation by DSC to obtain suitable boiling processing conditions for scallop (*Chlamys farreri*) and to reveal the mechanism behind the texture changes caused by boiling. To fulfill this goal, the changes in texture properties (hardness, chewiness, springiness, resilience, cohesiveness and shear force), sensory evaluation, physiochemical properties of proteins (myofibrillar protein (MP), water-soluble protein), amino acid composition of water-soluble proteins, water-soluble hydroxyproline (Hyp) content, protein aggregation, protein hydrophobicity, microstructure (under light microscopy (LM) and transmission electron microscopy (TEM)), and water-phase stage distribution (using low-field nuclear magnetic resonance (LF-NMR)) of the SAMs after boiling were investigated. Therefore, our findings offer a comparative study of different boiling-time effects on scallop texture and determine the boiling time by judging the protein denaturation by DSC. This will be of practical value from the perspective of food consumption for the improvement of quality on thermally processed products by controlled, moderate cooking.

## 2. Materials and Methods

### 2.1. Materials and Reagents

Fresh scallops (*Chlamys farreri*) with an average individual size of about 5–6.5 cm were purchased from the Qianhe local market in Dalian, Liaoning, China. The samples were kept at ice temperature and shipped immediately to the laboratory. All reagents used for gel electrophoresis were purchased from BioRad Co. (Hercules, CA, USA). The other reagents used in this work were analytical and purchased from Kemiou Chemical Reagent Co., Ltd. (Tianjin, China).

### 2.2. Boiling Processing

Adductor muscles with an average size of 1.5 cm were manually peeled from the scallop shells. After rinsing with distilled water and then draining the water, heat treatments of the scallop muscles were conducted in boiling distilled water (100 °C) (1:2, *w*/*v*) for 30 s, 3 min, and 15 min, respectively (the central temperature curve of SAMs measured by a core thermometer (Delta TRAK, Pleasanton, CA, USA) during boiling is shown in Figure S1). After the heat treatments, samples were immediately cooled in the ice-water until the core temperature reached below 3 °C. Next, a series of characterization experiments were performed, and the middle parts of the SAMs were cut for the histological analyses.

### 2.3. Thermal Transition Measurement

The endothermal transitions of samples were carried out by using a DSC III apparatus (Setaram Instrumentation, Caluire, France), as described in a previous study [11], with a few modifications. In brief, approximately 200 mg of minced samples (wet weight) in sample cell were heated in the range of 30 to 100 °C at the rate of 1 °C/min using the same weight of distilled water as a reference.

### 2.4. Shear Force and Texture Profile Analysis

The shear force and texture profile analysis (TPA) were carried out by using a TA-XT2i texture analyzer (Stable Micro Systems, Surrey, UK), according to a previous study [12]. SAM samples of uniform size (1.5 ± 0.1 cm) were selected for the shear force and TPA after cooling to room temperature (25 ± 1 °C) for 10 min. The shear force (muscle fibers of samples were vertically oriented to the blade) was assessed by using a HDP/BS (H = heavy duty platform/blade set) blade. The TPA (muscle fibers of samples were vertically oriented to the center of flat plate) was tested with a compression-cycle level of 60% at a pre-test speed 2.0 mm/s, test speed 1 mm/s, and post-test speed 1 mm/s using a P/5 probe.

### 2.5. Sensory Evaluation

Sensory evaluation of SAMs was carried out as described by Kulawik et al. [13], with some modifications. The evaluation of these samples was carried out by a panel of 20 judges for their sensory characteristics, such as appearance, flavor, texture, and taste. In this trial (blind), the judges were randomly given different scallop samples with random three-digit marks to hide their real names, and determined overall acceptability on the 7-point unipolar happiness scale, where 1 means completely unacceptable and 7 means perfect.

### 2.6. MP Extraction Rate

MPs from SAMs were extracted by 0.6 M NaCl solution, according to a previous study [12]. The extraction rate was the ratio of the amount of MP that was extracted from samples to their total protein content at the same mass.

### 2.7. Determination of Total Soluble Matter

The total soluble matter was calculated as follows:

Total soluble matter (g/100 g) = M_1_/M_2_ × 100 (1)

where M_1_ is dry weight of water-soluble fraction; M_2_ is dry weight of SAM used. The water-soluble fraction and SAM were oven dried under 105 °C until constant weight.

### 2.8. Water-Soluble Protein Content

A total of 10 g of SAM was homogenized with its boiling liquid in the ice-water bath by using an Ultra Turrax IKA T25 homogenizer (Staufen, Germany). After centrifugating at 10,500× *g* for 15 min at 4 °C, the supernatant was collected and diluted to 25 mL by distilled water for the further analysis. The content of water-soluble protein of SAM was expressed as the ratio of the amount of protein content in supernatant to its total protein content at the same mass. The content of protein in the supernatant was determined according to a previous study [14].

### 2.9. Determination of Amino Acid of Water-Soluble Fraction

Composition of total amino acid of previously extracted water-soluble supernatant was determined using a fully automatic amino analyzer (LA8080, Hitachi, Tokyo, Japan). The water-soluble fraction was hydrolyzed in 3 mL 6 M HCl at 110 °C for 22 h. Next, the sample was cooled and evaporated to dryness. Subsequently, the mixture was completely dissolved in 0.02 M HCl and filtered with a 0.22-micrometer membrane for use.

### 2.10. Sodium Dodecyl Sulfate Polyacrylamide Gel Electrophoresis

The sodium dodecyl sulfate polyacrylamide gel electrophoresis (SDS-PAGE) analysis was carried out according to a previous study [15]. SDS-PAGE analysis of MPs was performed by using 5% stacking gel and 12% separating gel, while that for water-soluble protein was 5% stacking gel and 10% separating gel.

### 2.11. Water-Soluble Hydroxyproline Content

The water-soluble supernatant was hydrolyzed in 6 M HCl at 130 °C for 4 h. Subsequently, the content of water-soluble Hyp in the supernatant was determined as described in a previous study [16], with L-Hyp as a standard, and was expressed as μg/g.

### 2.12. Fluorescence Microscopy

Protein aggregate was detected through microscopic techniques according to previous study [17]. Small blocks (1 cm^3^) were cut from the center of the SAMs and were frozen by liquid nitrogen. The frozen tissue blocks were sectioned at 10-micrometer thickness; next, samples were exposed for 3 h to Nile Red (0.04 µM, pH 6). Subsequently, sample sections were washed gently four times by phosphate buffer (pH 6.75) to remove unbound probe and observed under fluorescence microscope (Ti-s, Nikon Co., Tokyo, Japan) with a TRITC filter (the excitation and emission fluorescence ranges are 545–565 nm and 580–620 nm, respectively).

### 2.13. Light Microscopy

Small blocks (6 × 6 × 5 mm) were cut from the centers of the SAMs and fixed in 10% paraformaldehyde. Fixed samples were dehydrated with gradient concentrations of ethanol, cleared by xylene, and, finally, embedded by paraffin. The paraffin blocks (with sample tissue) were sectioned at 8-micrometer thickness, stained by hatmatoxylin-eosin (HE), and observed under the LM.

Protein hydrophobicity was detected through microscopic techniques, according to a previous study [17]. SAM was cut into small blocks (1 cm^3^) and frozen by liquid nitrogen. The frozen tissue blocks were sectioned at 10-micrometer thickness, after which samples were exposed for 1 h to bromophenol blue (BPB, 0.1 mg/mL, pH 6). Next, sample sections were washed gently four times by phosphate buffer (pH 6.75) to remove unbound probe and observed under the LM.

### 2.14. Transmission Electron Microscopy

The ultrastructure of SAMs of fresh and boiled samples were observed using a JEM-2000EX TEM (JEOL Ltd., Tokyo, Japan), as described in Liu et al. [18].

### 2.15. Water-Phase Distribution

The T2 transverse relaxation measurement and magnetic resonance imaging (MRI) analysis of samples were accomplished by low-field nuclear magnetic resonance, according to a previous study [19].

### 2.16. Statistical Analysis

One-way ANOVA and Duncan’s test were adopted to measure significant differences by using the software SPSS version 19.0 (SPSS Inc., Chicago, IL, USA). The results were expressed as mean ± standard deviation (SD). *p* values of <0.05 were considered statistically significant.

## 3. Results and Discussion

### 3.1. Determination of Boiling Conditions

Fresh SAMs were analyzed in boiling water for different durations, and the thermal behaviors of the boiled samples were determined by DSC analysis (Figure S2). Th thermal behavior of the fresh SAMs showed a typical thermogram with two endothermic transitions at 49.72 ± 0.10 °C (peak 1) and 72.26 ± 0.17 °C (peak 2), respectively (Figure S2). It was speculated that the first transition was mainly contributed by myosin and paramyosin, and the second transition was mainly contributed by actin [20]. With the increasing boiling time, the denaturation enthalpies (ΔH), estimated by measuring the area below the DSC transition curve, gradually decreased to zero (100 °C–3 min). Next, the two endothermic transitions disappeared, indicating that the proteins in the SAMs were completely denatured. The denaturation of the protein was used to determine whether the protein-based muscle foods were cooked [6,21]. The incomplete disappearance of the endothermic transition indicated that the proteins were not completely denatured; that is, the samples were undercooked. By contrast, samples with continuous cooking after moderate cooking were overcooked. Therefore, it can be inferred that the 30-s-boiled sample, 3-min-boiled sample and 15-min-boiled sample were undercooked, cooked and overcooked, respectively.

### 3.2. Changes in Proteins of Scallop Adductor Muscles

#### 3.2.1. Changes in Water-Soluble Proteins

**Figure 1:**
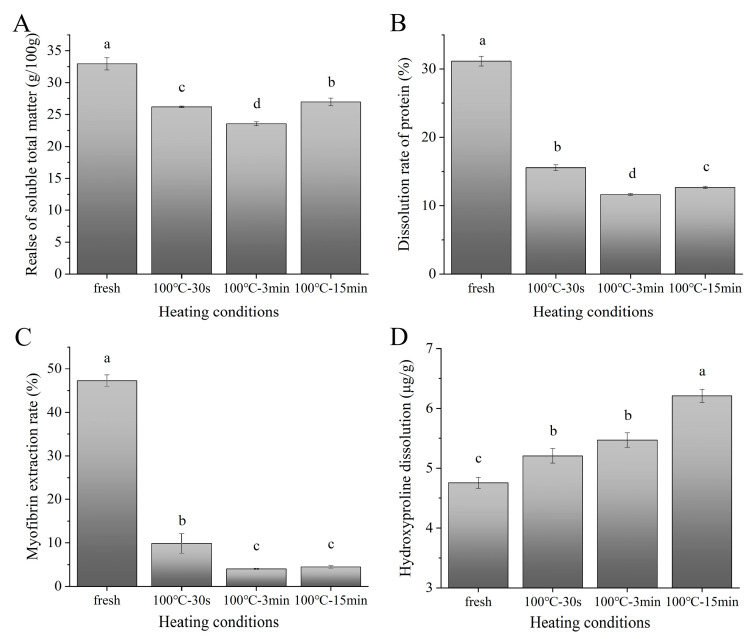
Changes in water-soluble matter (**A**), water-soluble protein (**B**), myofibrin extraction (**C**), and water-soluble Hyp (**D**) of SAMs after boiling. 100 °C–30 s, 30-s-boiled sample; 100 °C–3 min, 3-min-boiled sample; 100 °C–15 min, 15-min-boiled sample; values with different superscript letters indicate significant differences at the level of *p* < 0.05.

The changes in the water-soluble matter and water-soluble proteins of SAMs after boiling are shown in Figure 1A,B, respectively. The changing trend of the water-soluble matter was highly similar to that of the water-soluble proteins, which confirmed that the protein of the SAMs, which are protein-based muscle foods, was the main water-soluble component to have changed. All the boiled samples had significantly lower values than those of the fresh sample. This was due to the water-soluble protein aggregation, which decreased its solubility in the sample extracts. However, the 15-min-boiled sample released more water-soluble matter and water-soluble proteins than the 3 min-boiled sample, suggesting the degradation of water-insoluble proteins into water-soluble protein fragments. As shown in Figure S3A, the SDS-PAGE of the water-soluble proteins from the fresh SAMs showed nine major bands with a molecular weight (MW) in the range of 10 to 250 kDa. The boiling caused significant decreases in the intensities of the most of significant protein bands, which indicated their denaturation. The intensities of some bands (such as 35 kDa) showed time-dependent increasing trends, which were attributed to the progressive release of water-soluble protein fragments after boiling (Figure S3A). The total water-soluble amino acids also showed a trend of first decreasing and then increasing with the prolongation of the boiling time, which was consistent with the results of the water-soluble proteins (Table S1).

#### 3.2.2. Changes in Myofibrillar Proteins

The changes in the extraction rate of the MPs from the SAMs after boiling are shown in Figure 1C. The extraction rate of the SAMs decreased with the increasing boiling time, which was due to the denaturation of those salt-soluble proteins. As shown in Figure S3B, the SDS-PAGE of the MPs from the fresh SAMs mainly contained myosin heavy chain (MHC, 225 kDa), paramyosin (100 kDa), actin (43 kDa), and tropomyosin (35 kDa) [22]. The boiling caused significant decreases in the intensities of most of the major protein bands during the whole heat treatment, which might have been related to the protein denaturation and degradation. However, the boiling also caused the appearance of some low MW bands (ranging from 25 to 30 kDa), which might have been due to the degradation of the high-MW proteins. Thus, it can be seen that the components of the extractable salt-soluble MPs from the samples changed during cooking.

#### 3.2.3. Changes in Collagen

The changes in the water-soluble Hyp of the SAMs after boiling are shown in Figure 1D. They were determined to elucidate the degradation degree of the collagen [23]. The release in the water-soluble Hyp was noticed in the SAMs after boiling, which indicated the progressive degradation of the collagen. Similar results were reported by Wattanachant et al. [23], who found that the content of soluble collagen in chicken muscles increased during heat treatment. It is worth noting that the dissolution of Hyp increased significantly for the 15-min-boiled sample compared to the 3 min-boiled sample. Glycine and proline are the dominant amino acids in collagen [24]. Correspondingly, the characteristic amino acids (glycine and proline) of the collagen in the water-soluble fraction showed a significant increase in the 15 min-boiled sample (Table S1), which further proved the substantial degradation of the collagen.

### 3.3. Changes in the Protein Structure

**Figure 2:**
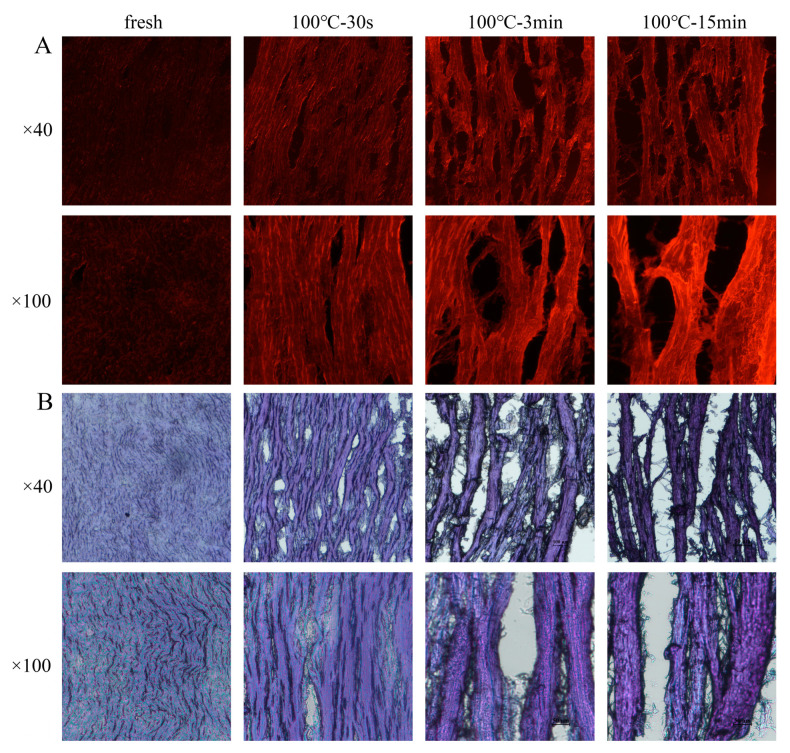
Fluorescence micrographs of longitudinal sections of SAMs after boiling stained by Nile red (**A**) and light micrographs of longitudinal sections of SAMs after boiling stained by bromophenol blue (**B**) at 40× and 100× field of view. 100 °C–30 s, 30-s-boiled sample; 100 °C–3 min, 3-min-boiled sample; 100 °C–15 min, 15-min-boiled sample.

To localize the changes in protein aggregation, microscopic observations of the SAMs stained by the Nile Red probe were performed, because protein aggregates containing hydrophobic surfaces bind Nile red more efficiently than monomers [17,25]. The fresh SAM exhibited a very low red fluorescence, whereas the boiled samples exhibited an increased red fluorescence with the extension of the boiling time (Figure 2A), which indicated the occurrence of protein aggregation. To localize the changes in protein hydrophobicity, microscopic observations of the SAMs stained by the BPB probe were also performed because the BPB can bind to hydrophobic groups on the surface of proteins [17]. The fresh SAM exhibited a lighter blue, whereas the boiled samples exhibited an increased darker blue, with the extension of the boiling time (Figure 2B), which indicated the enhanced protein hydrophobicity. Generally, the aggregation and increase in hydrophobicity of proteins during thermal processing can be attributed to protein denaturation and oxidative cross-linking [26].

### 3.4. Changes in Microstructure of Scallop Adductor Muscles

**Figure 3:**
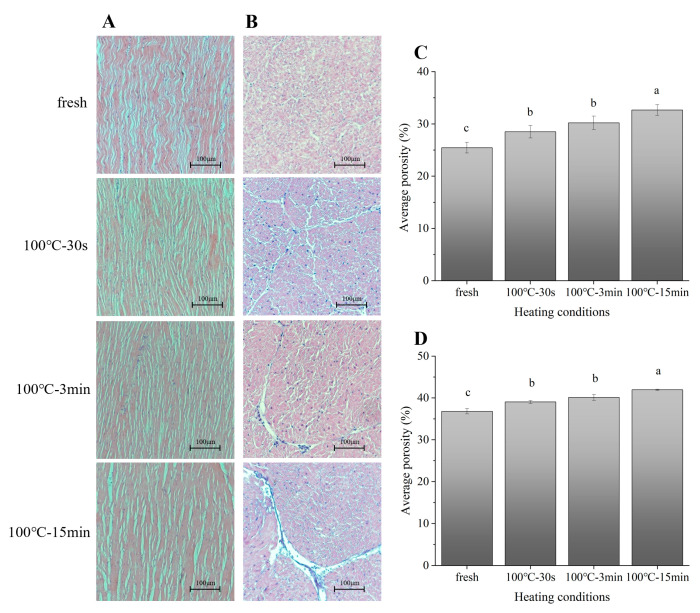
Light micrographs of longitudinal sections (**A**) and cross-sections (**B**) of SAMs after boiling stained by HE at 100× field of view. (**C**) Average porosity of longitudinal sections of samples; (**D**) Average porosity of cross-sections of samples; 100 °C–30 s, 30-s-boiled sample; 100 °C–3 min, 3-min-boiled sample; 100 °C–15 min-, 15-min-boiled sample; values with different superscript letters indicate significant differences at the level of *p* < 0.05.

Generally, the changes in the texture properties of muscle foods can be explained by the changes in muscle microstructure [27]. The LM of the longitudinal sections and cross-sections of the fresh and boiled SAMs stained by HE are shown in Figure 3A,B, respectively. According to the scale bar, it can be determined that the observed fibrous structures are muscle fibers, whose diameter should range from 10 to 100 μm [28]. The muscle fibers in the fresh sample were curved and evenly distributed, and the spaces between the muscle fibers were small and uniform. After boiling, the muscle fibers were straightened (Figure 3A). However, the overall length of the boiled SAMs decreased from the macroscopic perspective (Figure S4). Thus, it can be speculated that the boiling caused the longitudinal shrinkage of the muscle fibers. A previous study also found that the length of the sarcomere of bovine muscle decreased significantly with increasing heating temperature [29]. With the increase of the boiling time, the progressive aggregation of muscle fibers occurred, and the spaces around the assembled muscle-fiber clusters gradually increased. The quantitative histological parameters calculated by the software Image J also showed the increasing trends of horizontal and vertical porosities with the extension of the boiling time (Figure 3C,D). At the same time, the overall diameter of the boiled SAMs increased from the macroscopic perspective (Figure S4). Therefore, it was speculated that the increased spaces around the assembled muscle-fiber clusters may have contributed to the increased diameter of the boiled SAMs. In addition, the destruction of the connective tissue (including the perimysium and endomysium) between the muscle-fiber clusters in the 15-min-boiled sample was observed (Figure 3B), which also confirmed the degradation of the collagen [30,31]. Changes in muscle-protein structures and their spatial arrangement are often important factors that lead to changes in the texture of muscle foods [32]. The toughening effect of meat is usually accompanied by protein cross-linking, leading to the formation of muscle-protein aggregates, which has been demonstrated at the muscle-fiber level [33,34,35]. Therefore, the lateral cross-linking of muscle fibers caused by boiling processing may cause the increased toughening of SAMs. Additionally, connective tissue facilitates load sharing within muscle bundles by shearing connecting adjacent muscle fibers [36]. Therefore, the degradation of connective tissue caused by boiling processing may lead to dispersion between muscle-fiber bundles, resulting in a decrease in hardness and springiness.

**Figure 4:**
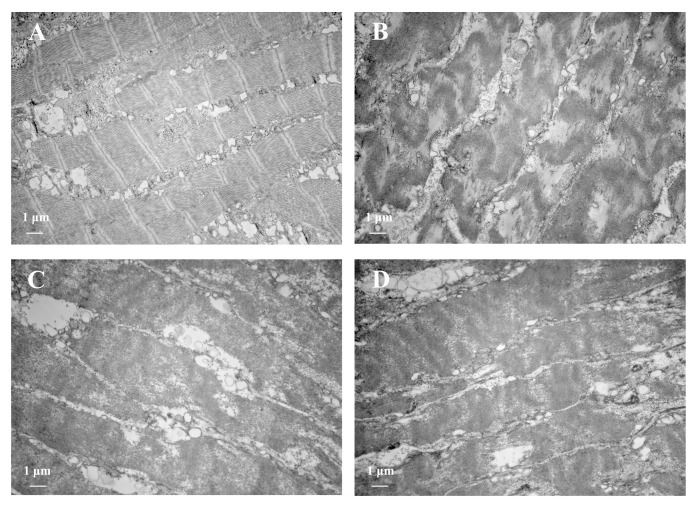
Transmission electron microscopy of myofibrils from SAMs after boiling at 12,000× field of view. (**A**) Fresh sample; (**B**) 30-s-boiled sample; (**C**) 3-min-boiled sample; (**D**) 15-min-boiled sample.

Myofibrils, the most important basic unit of muscle, can be observed in the TEM (Figure 4). The myofibrils of the fresh sample were dense, with a clear Z line and a relatively clear bright band (I zone) and dark band (A zone), which were surrounded by a dense and small sarcoplasmic reticulum (SR) [37]. After boiling, the Z line, I zone, and A zone of the myofibrils became unrecognizable. However, the outer contour of the myofibrils could still be clearly identified, although the SR between the myofibrils swelled. Compared with the fresh sample, the boiled samples showed a significant increase in myofibril diameter, which was due to the lateral expansion caused by the longitudinal contraction of the myofibrils [38]. It was pointed out previously that the toughening of meat products at high temperatures may be related to the longitudinal shrinkage of myofibrillar components [36].

### 3.5. Changes in Water-Phase Distribution

**Figure 5:**
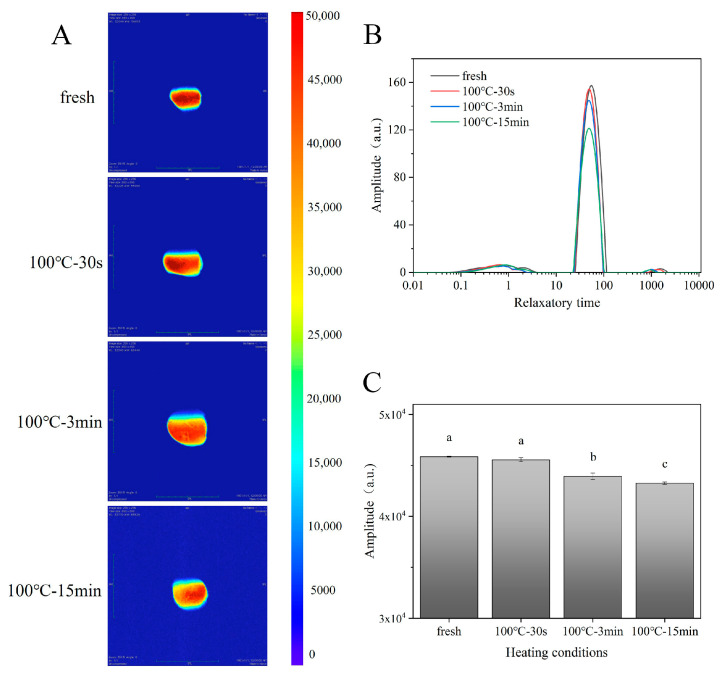
Changes in water-weighted magnetic resonance images (MRI). (**A**) *T*_2_ relaxation time; (**B**) the quantitative signal intensity of MRI (**C**) of SAM after boiling. 100 °C–30 s, 30-s-boiled sample; 100 °C–3 min, 3-min-boiled sample; 100 °C–15 min, 15-min-boiled sample; values with different superscript letters indicate significant differences at the level of *p* < 0.05.

Variance in the water-phase state and distribution may be involved in the changes of texture of cooked muscle food [32]. Hence, T2 relaxation spectra by multi-exponential fitting and water-weighted MRI imaging, with the corresponding relative intensity of the SAMs after boiling, were obtained (Figure 5 and Table S2). In the T2 relaxation spectra of the SAMs, three water populations, defined as T21, T22, and T23 were observed (Figure 5B), which could be assigned as bound water, immobile water, and free water, respectively. For muscle tissues, immobile water mainly occurs in the myofibril network inside the muscle cells, while water that can flow freely mainly occurs in the interstitial space outside the muscle cells [39]. The percentages of bound water, immobile water, and free water for the fresh samples were 5.54%, 93.71%, and 0.75%, respectively, according to the peak-area normalization. Along with the extension of the boiling time, the peak area of the immobile water decreased gradually, but that of the free water increased gradually (Table S2). At the same time, the water-weighted MRI images and their quantitative analysis also showed a declining trend, in contrast to the intensity with the prolonged boiling time, indicating the progressive loss of water during boiling (Figure 5). These indicated that the boiling processing resulted in part of the immobile water being converted to free water, as well as the loss of free water. Compared with the fresh sample, the relaxation time T22 of the boiled samples also decreased gradually, along with the extension of the boiling time (Figure 5 and Table S2). The relaxation time reflected the chemical environment of the hydrogen protons inside the muscle sample, and was related to the binding force and the freedom degree of the hydrogen protons. The shorter the relaxation time, the greater the bondage of hydrogen protons or the lower freedom degree [40]. Therefore, it was speculated that the shrinkage of the muscle cells after boiling processing squeezed out the immobile water in the myofibril network inside to the interstitial space outside, becoming free water, after which the retained immobile water had a greater hydrogen bond energy, resulting in a shortened relaxation time T22 [41]. Previous studies indicated that the extrusion of immobile water into free water and the loss of free water caused by heat treatment could lead to the toughening of meat products [40,42,43].

### 3.6. Changes in Texture Properties and Their Mechanism

**Figure 6:**
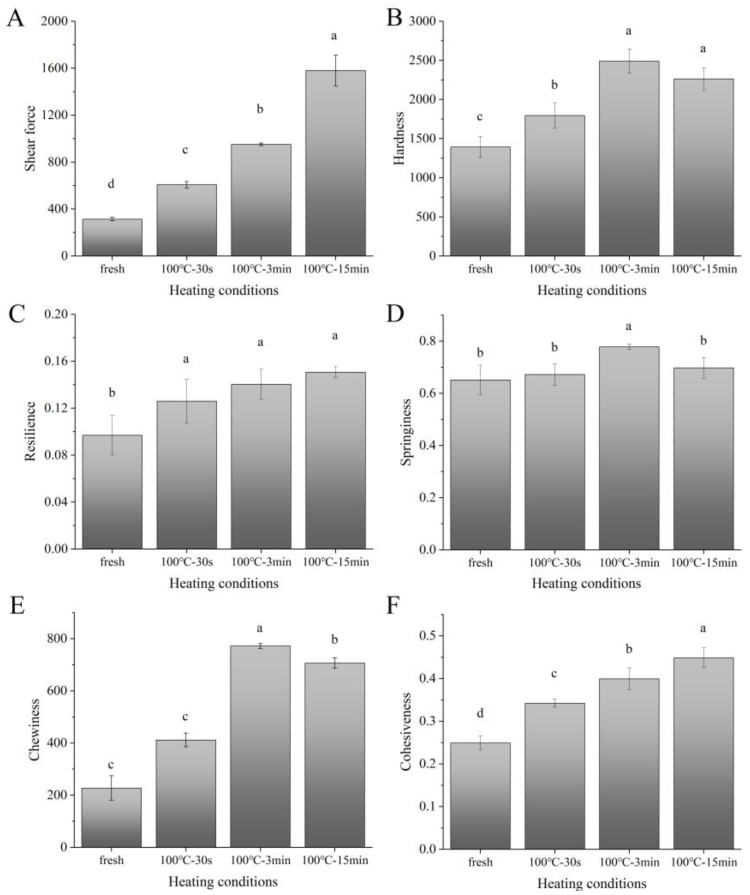
Changes in shear force (**A**), hardness (**B**), resilience (**C**), springiness (**D**), chewiness (**E**), and cohesiveness (**F**) of SAM after boiling. 100 °C–30 s, 30-s-boiled sample; 100 °C–3 min, 3-min-boiled sample; 100 °C–15 min, 15-min-boiled sample; values with different superscript letters indicate significant differences at the level of *p* < 0.05.

The effects of different boiling times on the texture properties, including hardness, chewiness, springiness, resilience, cohesiveness and shear force, of the boiled SAMs are shown in Figure 6. Compared to the fresh sample, all the indices of the texture properties of the boiled SAMs increased significantly (*p* < 0.05), which is a common characteristic in muscle foods after cooking [44]. Our results showed that the boiling processing of the SAMs led to the denaturation, aggregation, and increased hydrophobicity of the proteins, causing the longitudinal contraction and lateral expansion of the myofibrils, the longitudinal contraction and lateral cross-linked aggregation of the muscle fibers, and the consequent extrusion of the immobile water, as well as the loss of free water. These may be the reasons for the rise in the overall texture indicators of the boiled samples compared to the fresh sample.

Up to 3 min of boiling, all the texture indicators of the SAMs increased time-dependently (Figure 6), which not only did not produce a negative chewy feel due to the tougher texture, but also increased the springiness to a certain extent, improving to the results on the sensory evaluation (Table S3). This is different from meat products, such as beef, pork, and chicken, which easily form a tougher texture after heat treatment, resulting in poor sensory experiences [6,44]. For muscle foods, the higher the collagen content, the greater the shear force and hardness [45]. The muscles of small aquatic animals, such as fish and shellfish, generally have lower amounts of collagen compared to larger terrestrial animals [28], giving them a softer texture. In addition, Ertbjerg and Puolanne [46] suggested that for muscle foods, the toughening was due to the muscle contraction caused by heating-induced shortened sarcomere length. Our results showed that the shrinkage of the sarcomere length in the SAMs after boiling processing was about 20–35%, according to the longitudinal contraction of the muscle tissue, while that of beef can reach more than 60% [46]. Therefore, the difference in the collagenous tissue content and the shrinkage of the sarcomere length after cooking may be the reasons for the differences in the texture properties between cooked SAMs and cooked meat products, such as beef, pork and chicken.

As the boiling time increased to 15 min, the shear force and cohesiveness of the SAMs still increased significantly (*p* < 0.05), and the resilience and hardness were maintained (*p* > 0.05), but the springiness and chewiness decreased significantly (*p* < 0.05). According to their appearance, the muscle fibers of the 15-min-boiled SAMs tended to scatter into muscle-fiber bundles (Figure S5). From the sensory perspective, the 15-min-boiled sample showed pronounced frangibility between the muscle fibers, but the fibers became tougher, resulting in a decreased sensory texture score (Table S3). Our results also showed that 15 min of boiling caused the significant degradation of the proteins (especially collagen) in the SAMs, resulting in the destruction of the connective tissues (including perimysium and endomysium) between the muscle-fiber clusters, which may be the reason for the texture deterioration of the 15-min-boiled sample.

## 4. Conclusions

The present study revealed the mechanism behind the effects of boiling processing on textural properties. Boiling induced protein denaturation, aggregation, and increased hydrophobicity, leading to the longitudinal contraction and lateral expansion of myofibrils, the longitudinal contraction and lateral cross-linked aggregation of muscle fibers, as well as the loss of free water, which may have contributed to the overall increasing texture properties of the boiled SAMs. However, the significant degradation of the proteins (especially collagen) caused by 15 min of boiling destroyed the connective tissue between the muscle-fiber clusters, which may have contributed to the decreasing springiness and chewiness of the boiled SAMs. Therefore, we recommended 3-min-boiled SAMs, both from a subjective sensory point of view and from the objective point of view of protein denaturation and degradation. In future, key proteins that affect the formation of the textural properties of cooked SAMs should be determined to further reveal its deeper mechanism.

## Data Availability

Data is contained within the article or supplementary material.

## Supplementary Materials

The following supporting information can be downloaded at: https://www.mdpi.com/article/10.3390/foods11131947/s1. Figure S1: The central temperature curve of SAMs during boiling. Figure S2: Changes in the differential scanning calorimetry of SAMs after boiling. 30 s, 30-s-boiled sample; 2.5 min, 2.5-min-boiled sample; 3 min, 3-min-boiled sample; 15 min, 15-min-boiled sample. Figure S3: The sodium dodecyl sulfate polyacrylamide gel electrophoresis of water-soluble protein (A) and myofibrillar protein (B) of SAMs after boiling. Samples 1–4 stand for fresh sample, 30-s-boiled sample, 3-min-boiled sample, and 15-min-boiled sample, respectively. MHC, myosin heavy chain. Figure S4: The changes in length (A) and diameter (B) of SAMs after boiling. 100–30 s, 30-s-boiled sample; 100–3 min, 3-min-boiled sample; 100–15 min, 15-min-boiled sample; values with different superscript letters indicate significant differences at the level of *p* < 0.05. Figure S5: Changes in appearance of muscle tissue of SAMs after boiling. 30 s, 30-s-boiled sample; 3 min, 3-min-boiled sample. 15 min, 15-min-boiled sample. Table S1: Changes in amino acids of water-soluble fractions from SAMs after boiling (mg/g dry basis). Table S2: Changes in water distribution of SAMs after boiling. Table S3: Sensory evaluation of SAMs after boiling.
