# Supplementary material for: Effects of Boiling Processing on Texture of Scallop Adductor Muscle and Its Mechanism
Source: Foods. 2022 Jun 30;11(13):1947. doi: 10.3390/foods11131947 (PMC9265745; doi:10.3390/foods11131947)
Supplement: Supplementary file 1 [file foods-11-01947-s001.zip › Figure S3.pdf]

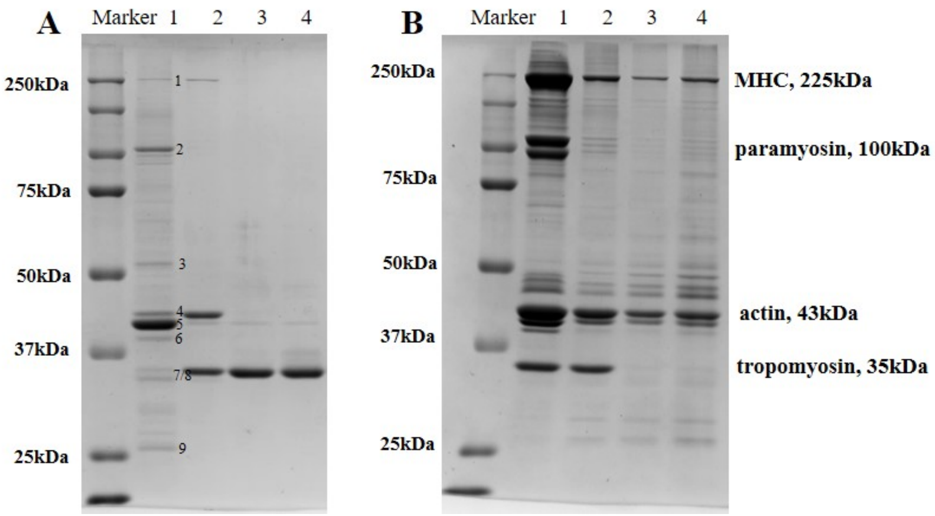

Figure S3. The sodium dodecyl sulfate polyacrylamide gel electrophoresis of water-soluble protein (A) and myofibrillar protein (B) of SAMs during boiling. Sample 1-4 stand for fresh sample, 30 s-boiled sample, 3 min-boiled sample and 15 min-boiled sample, respectively. MHC, myosin heavy chain.
