# Supplementary material for: Effects of Boiling Processing on Texture of Scallop Adductor Muscle and Its Mechanism
Source: Foods. 2022 Jun 30;11(13):1947. doi: 10.3390/foods11131947 (PMC9265745; doi:10.3390/foods11131947)
Supplement: Supplementary file 1 [file foods-11-01947-s001.zip › Figure S4.pdf]

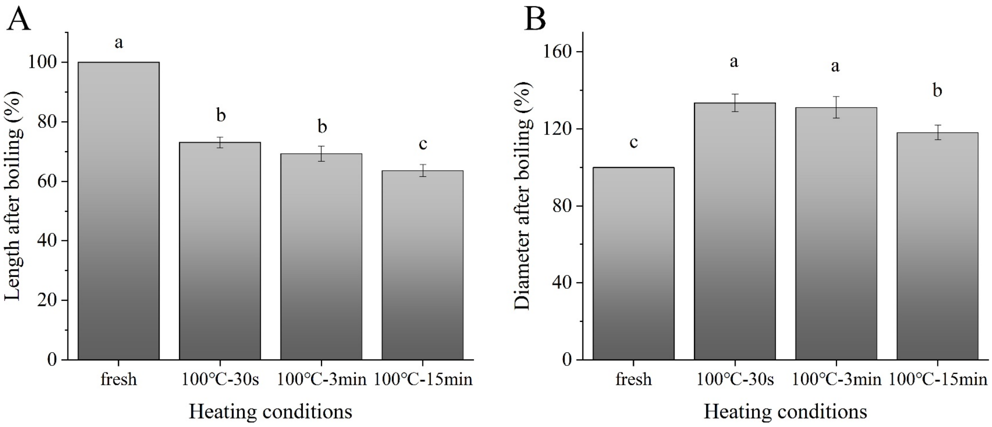

Figure S4. The changes of length (A) and diameter (B) of SAMs during boiling. 100 ℃-30 s, 30 s-boiled sample; 100 ℃-3 min, 3 min-boiled sample; 100 ℃-15 min, 15 min-boiled sample; values with different superscript letters indicate significant differences at the level of $P < 0.05$.
