# Supplementary material for: Effects of Boiling Processing on Texture of Scallop Adductor Muscle and Its Mechanism
Source: Foods. 2022 Jun 30;11(13):1947. doi: 10.3390/foods11131947 (PMC9265745; doi:10.3390/foods11131947)
Supplement: Supplementary file 1 [file foods-11-01947-s001.zip › Figure S5.pdf]

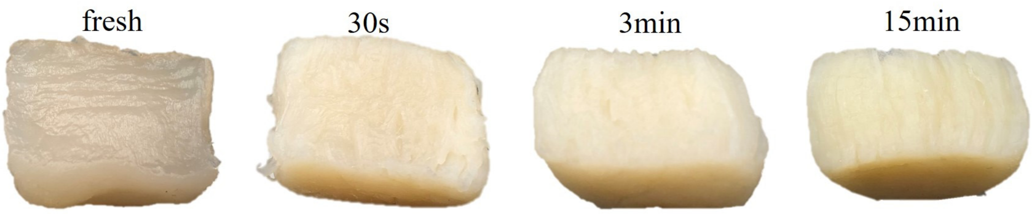

Figure S5. Changes in appearance of muscle tissue of SAMs during boiling. 30 s, 30 s-boiled sample; 3 min, 3 min-boiled sample; 15 min, 15 min-boiled sample.
