# Supplementary material for: Effects of Boiling Processing on Texture of Scallop Adductor Muscle and Its Mechanism
Source: Foods. 2022 Jun 30;11(13):1947. doi: 10.3390/foods11131947 (PMC9265745; doi:10.3390/foods11131947)
Supplement: Supplementary file 1 [file foods-11-01947-s001.zip › Table S1.pdf]

Table S1.

Changes in amino acids of water-soluble fractions from SAMs during boiling (mg/g dry basis).

| mg/g dry basis | Fresh | 100 °C-30 s | 100 °C-3 min | 100 °C-15 min |
|---|---|---|---|---|
| Asp | 9.34±0.21a | 5.34±0.22b | 4.73±0.14c | 5.17±0.09b |
| Thr | 3.80±0.10a | 2.30±0.16b | 1.75±0.07d | 1.98±0.04c |
| Ser | 3.20±0.11a | 1.73±0.18b | 1.11±0.05d | 1.29±0.03c |
| Glu | 10.61±0.21a | 9.07±0.58b | 8.96±0.16b | 10.23±0.14a |
| Gly | 30.20±0.70b | 30.46±1.04b | 33.51±1.16a | 34.2±0.61a |
| Ala | 7.86±0.10a | 5.3±0.11d | 3.59±0.19c | 4.87±0.08b |
| Val | 6.29±0.29a | 2.05±0.02b | 0.98±0.03d | 1.37±0.04c |
| Met | 1.26±0.04 | nd | nd | nd |
| lle | 2.55±0.11a | 1.14±0.05b | 0.55±0.03c | 0.59±0.02c |
| Leu | 3.58±0.16a | 1.98±0.13b | 1.75±0.13c | 1.87±0.09bc |
| Tyr | 2.96±1.38a | 2.11±0.31a | 1.84±0.45a | 1.88±0.41a |
| Phe | 5.11±0.87a | 2.73±0.43b | 2.56±0.27b | 2.75±0.31b |
| Lys | 5.26±0.15a | 2.39±0.11bc | 2.27±0.11c | 2.49±0.06b |
| His | 2.15±0.07a | 0.71±0.05b | 0.34±0.03c | 0.35±0.01c |
| Arg | 13.90±0.54c | 17.41±0.79b | 18.48±0.86ab | 19.34±0.52a |
| Pro | 14.90±0.33a | 12.4±0.51c | 12.08±0.68c | 13.96±0.29b |
| Total amino acids | 122.98±0.02a | 97.15±4.63c | 94.50±2.40c | 102.35±1.40b |
| Collagen-related amino acids | 45.10±1.03b | 42.87±1.55b | 45.59±1.40b | 48.16±0.77a |

100 °C-30 s, 30 s-boiled sample; 100 °C-3 min, 3 min-boiled sample; 100 °C-15 min, 15 min-boiled sample; nd, not detected; Values with different superscript letters indicate significant differences at the level of $P < 0.05$.
