# Supplementary material for: Effects of Boiling Processing on Texture of Scallop Adductor Muscle and Its Mechanism
Source: Foods. 2022 Jun 30;11(13):1947. doi: 10.3390/foods11131947 (PMC9265745; doi:10.3390/foods11131947)
Supplement: Supplementary file 1 [file foods-11-01947-s001.zip › Table S2.pdf]

Table S2.

Changes in water distribution of SAMs during boiling.

Samples	Fresh	100 °C-30 s	100 °C-3 min	100 °C-15 min
T_{21}(ms)	0.93±0.06a	0.69±0.05c	0.77±0.01c	0.88±0.03b
T_{22}	55.51±0.89a	50.03±3.50b	49.46±0.80c	47.25±1.52c
T_{23}	1524.11±24.73a	1422.13±23.08b	1066.54±46.42c	962.49±64.22d
AT_{21}	390.04±7.99a	404.43±15.43a	410.59±13.30a	402.59±15.69a
AT_{22}	6594.33±168.44a	6053.99±102.57b	5817.59±137.26c	5381.84±110.70d
AT_{23}	52.70±3.25c	56.24±1.65c	60.12±0.83b	64.61±1.77a
P_{21}(%)	5.54±0.13c	6.21±0.24b	6.53±0.17ab	6.89±0.32a
P_{22}	93.71±0.16a	92.93±0.23b	92.51±0.17c	92.01±0.32d
P_{23}	0.75±0.04d	0.86±0.03c	0.96±0.03b	1.10±0.04a

100 °C-30 s, 30 s-boiled sample; 100 °C -3 min, 3 min-boiled sample; 100 °C -15 min, 15 min-boiled sample, values with different superscript letters indicate significant differences at the level of $P < 0.05$.
