# Supplementary material for: Effects of Boiling Processing on Texture of Scallop Adductor Muscle and Its Mechanism
Source: Foods. 2022 Jun 30;11(13):1947. doi: 10.3390/foods11131947 (PMC9265745; doi:10.3390/foods11131947)
Supplement: Supplementary file 1 [file foods-11-01947-s001.zip › Table S3.pdf]

Table S3.

Sensory evaluation of SAMs during boiling.

Sample	Fresh	100 °C-30 s	100 °C-3 min	100 °C-15 min
appearance	6.56±0.63a	5.13±1.41ab	4.94±1.12b	4.75±1.53b
flavor	2.00±1.26b	3.75±1.53ab	5.44±1.15a	5.63±0.96a
texture	3.28±1.25b	4.48±1.48ab	6.45±1.03a	5.25±0.79a
taste	2.06±1.73b	3.13±1.20b	5.63±1.26a	5.25±0.84a
Overall acceptance	2.00±1.46b	3.00±1.59b	5.63±1.02a	5.25±1.61ab

100 °C-30 s, 30 s-boiled sample; 100 °C-3 min, 3 min-boiled sample; 100 °C-15 min, 15 min-boiled sample.
