# Supplementary figures and images for: Effects of Boiling Processing on Texture of Scallop Adductor Muscle and Its Mechanism
Source: Foods. 2022 Jun 30;11(13):1947. doi: 10.3390/foods11131947 (PMC9265745; doi:10.3390/foods11131947)

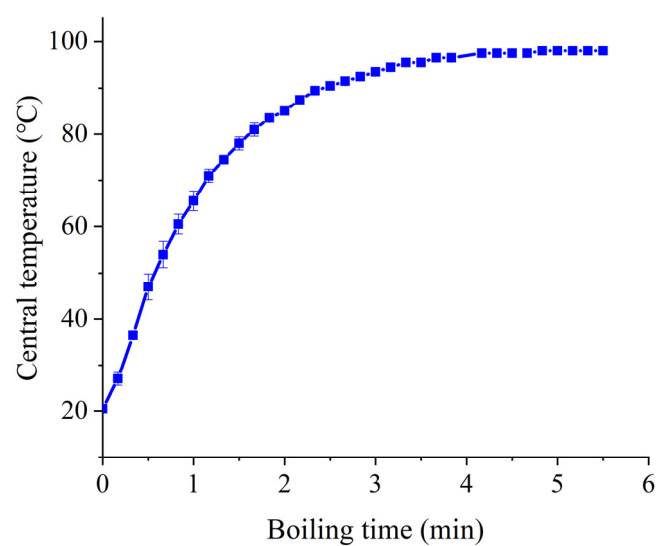

Figure S1. The central temperature curve of SAMs during boiling.

Supplement: Supplementary file 1 [file foods-11-01947-s001.zip › Figure S1.pdf]
